# Round-the-clock performance of coronary CT angiography for suspected acute coronary syndrome: Results from the BEACON trial

**DOI:** 10.1007/s00330-017-5082-7

**Published:** 2017-12-15

**Authors:** Marisa M. Lubbers, Admir Dedic, Akira Kurata, Marcel Dijkshoorn, Jeroen Schaap, Jeroen Lammers, Evert J. Lamfers, Benno J. Rensing, Richard L. Braam, Hendrik M. Nathoe, Johannes C. Post, Pleunie P. Rood, Carl J. Schultz, Adriaan Moelker, Mohamed Ouhlous, Bas M. van Dalen, Eric Boersma, Koen Nieman

**Affiliations:** 1 Department of Cardiology, Erasmus University Medical Center, Room: Ca-207a, ‘s-Gravendijkwal 230, Rotterdam, 3015 CE The Netherlands; 2 Department of Radiology, Erasmus University Medical Center, ‘s-Gravendijkwal 230, 3015 CE Rotterdam, The Netherlands; 3 Department of Cardiology, Amphia Ziekenhuis, Molengracht 21, 4818 CK Breda, The Netherlands; 4 Department of Cardiology, Elkerliek Ziekenhuis, Wesselmanlaan 25, 5707 HA Helmond, The Netherlands; 5 Department of Cardiology, Canisius Wilhelmina Ziekenhuis, Weg door Jonkerbos 100, 6532 SZ Nijmegen, The Netherlands; 6 Department of Cardiology, St Antonius Ziekenhuis, Koekoekslaan 1, 3435 CM Nieuwegein, The Netherlands; 7 Department of Cardiology, Gelre Ziekenhuis, Albert Schweitzerlaan 31, 7334 DZ Apeldoorn, The Netherlands; 8 Department of Cardiology, University Medical Center Utrecht, Heidelberglaan 100, 3584 CX Utrecht, The Netherlands; 9 Department of Emergency Medicine, Erasmus University Medical Center, ‘s-Gravendijkwal 230, 3015 CE Rotterdam, The Netherlands; 10 Department of Cardiology, Royal Perth Hospital Campus, University of Western Australia, Perth, WA 6000 Australia; 11 Stanford Cardiovascular Institute, Stanford University, 265 Campus Drive, Stanford, CA 94305 USA

**Keywords:** Acute coronary syndrome, Coronary artery disease, Coronary CT angiography, Emergency department, Image quality

## Abstract

**Objective:**

To assess the image quality of coronary CT angiography (CCTA) for suspected acute coronary syndrome (ACS) outside office hours.

**Methods:**

Patients with symptoms suggestive of an ACS underwent CCTA at the emergency department 24 hours, 7 days a week. A total of 118 patients, of whom 89 (75 %) presented during office hours (weekdays between 07:00 and 17:00) and 29 (25 %) outside office hours (weekdays between 17:00 and 07:00, weekends and holidays) underwent CCTA. Image quality was evaluated per coronary segment by two experienced readers and graded on an ordinal scale ranging from 1 to 3.

**Results:**

There were no significant differences in acquisition parameters, beta-blocker administration or heart rate between patients presenting during office hours and outside office hours. The median quality score per patient was 30.5 [interquartile range 26.0–33.5] for patients presenting during office hours in comparison to 27.5 [19.75–32.0] for patients presenting outside office hours (p=0.043). The number of non-evaluable segments was lower for patients presenting during office hours (0 [0–1.0] vs. 1.0 [0–4.0], p=0.009).

**Conclusion:**

Image quality of CCTA outside office hours in the diagnosis of suspected ACS is diminished.

***Key Points*:**

• *Quality scores were higher for coronary-CTA during office hours.*

• *There were no differences in acquisition parameters.*

• *There was a non-significant trend towards higher heart rates outside office hours.*

• *Coronary-CTA on the ED requires state-of-the-art scanner technology and sufficiently trained staff.*

• *Coronary-CTA on the ED needs preparation time and optimisation of the procedure.*

## Introduction

The optimal diagnostic work-up of suspected acute coronary syndrome (ACS) remains a topic of controversy [1]. Recently, several trials have demonstrated that coronary CT angiography (CCTA) is a safe and potentially more efficient diagnostic option for the triage of patients with acute chest pain [2–7]. While patients with acute chest pain may seek medical attention at any time during the day, most medical centres offer CCTA only during office hours. A round-the-clock CCTA service poses various challenges, in terms of the availability of scanners and experienced staff, as well as patient characteristics and severity of disease [8, 9]. It is currently unknown whether the time of the day affects the image quality of CCTA. In this pre-specified sub-analysis of the Better Evaluation of Acute Chest Pain with Computed Tomography Angiography (BEACON) trial, we investigated the feasibility of CCTA outside office hours.

## Materials and methods

### Study design and participants

In the multicentre randomised BEACON trial, we compared a diagnostic strategy with early CCTA to standard optimal care in patients suspected of acute coronary syndrome. At one institution, patients were enrolled 24 hours, 7 days a week. The study design, inclusion and exclusion criteria, and primary results have been reported previously [5]. Briefly, we enrolled patients with symptoms suggestive of an ACS at the emergency department (ED). Exclusion criteria included the need for urgent cardiac catheterisation and history of ACS or coronary revascularisation.

Study participants were divided into patients presenting during regular office hours (weekdays between 07:00 and 17:00) and outside office hours (weekdays between 17:00 and 07:00, weekends and holidays). Information regarding clinical characteristics, time of presentation, time between presentation and CT angiography, image quality, presence of coronary artery disease and clinical outcome were collected prospectively.

### Procedures

After initial workup at the ED, consisting of a clinical evaluation including an ECG and laboratory tests, patients underwent CCTA. Image acquisition was performed on either a 128-slice single source scanner, present at the ED or a dual-source system, situated at the radiology department depending on physician preference and availability. We used ECG-synchronised axial or spiral scan protocols combined with radiation-minimising measures. During the day CT scans were performed by a cardiac dedicated technician team. At night the examinations were performed by a broader group of technicians, although all were trained to perform cardiac CT. If indicated and clinically acceptable, beta-blockers were administrated. All patients received sublingual nitroglycerin 1 min before scanning. First a coronary calcium scan was performed, and the Agatston calcium score calculated. Subsequent CCTA was evaluated according to SCCT criteria [10]. For clinical decision making CCTAs were assessed at the point of care by cardiologists and radiologists with at least 5 years of experience in cardiac CT.

### Image evaluation

Image quality was evaluated per segment in accordance with the American Heart Association (AHA) classification [10], by two independent observers (A.K. and A.D., each with more than 3 years’ experience), blinded to the time of acquisition. Data sets were transferred offline and evaluated for quality purposes at a later time. If necessary, multiple data sets were used for the quality assessment in sequential and retrospective spiral scans. Artefacts were defined as stack, motion, breathing, blooming, noise or streak. Small segments with a diameter of less than 1.5 mm were excluded from analysis. Image quality was graded on an ordinal scale ranging from 1 to 3. Segments scored as 1 represented poor image quality due to major artefacts and diagnostic evaluation was deemed impossible. In segments scored as 2 there were artefacts present, but image quality was adequate for diagnostic evaluation, and segments scored as 3 had no artefacts with good image quality. Image quality was assessed for all segments of each coronary artery (left main coronary artery, left anterior descending coronary artery, left circumflex coronary artery and right coronary artery) and then averaged for every patient. Image quality comparison was performed using several approaches: (1) the median quality score per patient; (2) the median quality score per patient of the proximal coronary segments (segments 1, 2, 5-7 and 11); (3) the number of non-evaluable segments per patient; and (4) the number of non-evaluable proximal coronary segments per patient.

Values for effective radiation dose (mSv) were calculated by multiplying the dose-length product (DLP) with a conversion factor for cardiac CT of κ = 0.017 mSv/mGy x cm [11].

### Statistical analysis

Continuous data are presented as means ± SD or medians with interquartile ranges as appropriate, and categorical variables as frequencies or percentages. Groups were compared using an independent-sample t-test or Mann-Whitney U-test for continuous variables, and chi-square or Fisher’s exact-test for categorical variables. A two-sided p-value of <0.05 was considered statistically significant. Statistical analyses were performed using SPSS (version 21, IBM Corp, Armonk, NY, USA).

## Results

### Study population

A total of 118 patients with suspected acute coronary syndrome underwent CCTA. Eighty-nine (75 %) presented during office hours and 29 (25 %) outside office hours. Baseline characteristics are shown in Table 1. The mean age was 54 ± 10, 47 % were women and the majority had a low GRACE risk score. Aside from ischaemic ECG abnormalities (mostly T-wave abnormalities), which were seen more frequently in patients presenting during office-hours, no differences were found in other baseline characteristics.

**Table 1:** Baseline characteristics

	Office hours (n=89)	Outside office hours (n=29)	p-value
Age, years	54 ± 10	55± 11	0.749
Sex, female	42 (47)	15 (52)	0.671
Blood pressure, systolic, mm Hg	140 ± 16	143 ± 16	0.332
Blood pressure, diastolic, mm Hg	84 ± 12	86 ± 10	0.456
Cardiovascular risk factors			
Diabetes mellitus	8 (9)	3 (10)	0.330
Hypercholesterolaemia	25 (28)	10 (34)	0.612
Smoking	41 (46)	18 (62)	0.187
Family history	34 (38)	13 (45)	0.663
Hypertension	39 (44)	14 (48)	0.798
TIMI risk score	1 (0-2)	1 (1-2)	0.259
0	29 (33)	5 (17)	
1	33 (37)	12 (41)	
≥ 2	27 (30)	12 (41)	
Grace risk score	85 [70–98]	96 [72–121]	0.131
Low	76 (85)	21 (72)	
Intermediate	12 (13)	6 (21)	
High	1 (1)	2 (7)	
Ischaemic ECG abnormalities	26 (29)	15 (52)	0.042
Baseline hs troponins elevated^a^	36 (40)	13 (45)	0.829

Values are mean ± SD, n (%) or median [interquartile range]

Diabetes mellitus is defined as plasma glucose > 11.0 mmol/L or treated with either diet regulation or medication. Hypertension is defined as > 150 mm Hg systolic or > 90 mm Hg diastolic or treated with medication. Ischaemic ECG abnormalities are defined as Q-wave or ST-T segment alterations suggestive of ischaemia.

^a^Elevated within three times the upper limit of the 99th percentile

**Fig. 1:**
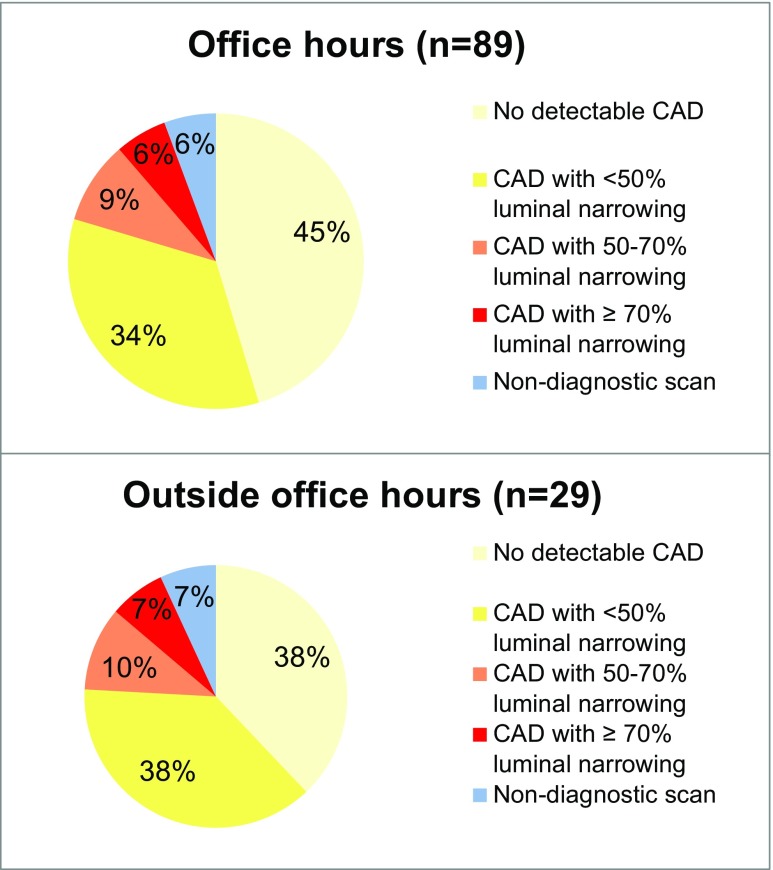
CT results stratified for patients presenting during and outside office hours. All p > 0.05. *CAD* coronary artery disease

The median Agatston calcium score was 1 [0–30] in patients presenting during office hours and 4 [0–48] in patients presenting outside office hours. CCTA identified 40 (45 %) patients presenting during office hours with no detectable CAD, compared to 11 (38 %) patients presenting outside office hours. Obstructive CAD (> 50 % luminal narrowing) was found in 13 (15 %) patients presenting during office hours and five (17 %) presenting outside office hours. The scan was considered non-diagnostic in five (6 %) patients presenting during office-hours and in two (7 %) patients presenting outside office hours (all p > 0.05) (Fig. 1). The mean radiation dose for CT angiography was 4.2 ± 3.7 mSv for patients presenting during office hours and 4.2 ± 2.8 mSv for patients presenting outside office hours (p=0.957).

### CT acquisition

Scan acquisition parameters are shown in Table 2. There were no significant differences in acquisition parameters between patients presenting during office hours and outside office hours. The number of patients receiving intravenous beta-receptor antagonists prior to the scan was similar between the office and outside-office hours presenting patients, as well was the dose (Table 2). The mean heart rate was higher in the group presenting outside office hours, although it did not reach statistical significance (64 ± 13 vs. 69 ± 13, p=0.095).

**Table 2:** Scan acquisition parameters

	Office hours (n=89)	Outside office hours (n=29)	p-value
Type CT scanner			0.340
Single source 128-slice	67 (75)	19 (66)	
Dual source 128-slice	22 (25)	10 (34)	
Heart rate/min. during scanning (range)	64 ± 13 (38–100)	69 ± 13 (50–98)	0.095
Scan protocol			0.183
Axial	76 (85)	28 (97)	
Spiral	13 (15)	1 (3)	
Beta blocker administration			0.590
Yes	47 (53)	13 (45)	
No	19 (21)	7 (24)	
Missing	23 (26)	9 (31)	
Beta blocker dose	5 [5–6.5]	5 [5–6.25]	0.371
Tube voltage, kV	100 [100–120]	120 [100–120]	0.361
Tube current-time product, mAs	194 [144–332]	322 [146–307]	0.562
Dose length product (DLP)	280 ± 245	271 ± 198	0.851

Values are n (%), median [interquartile range], or means ± standard deviation

### Image quality

**Fig. 2:**
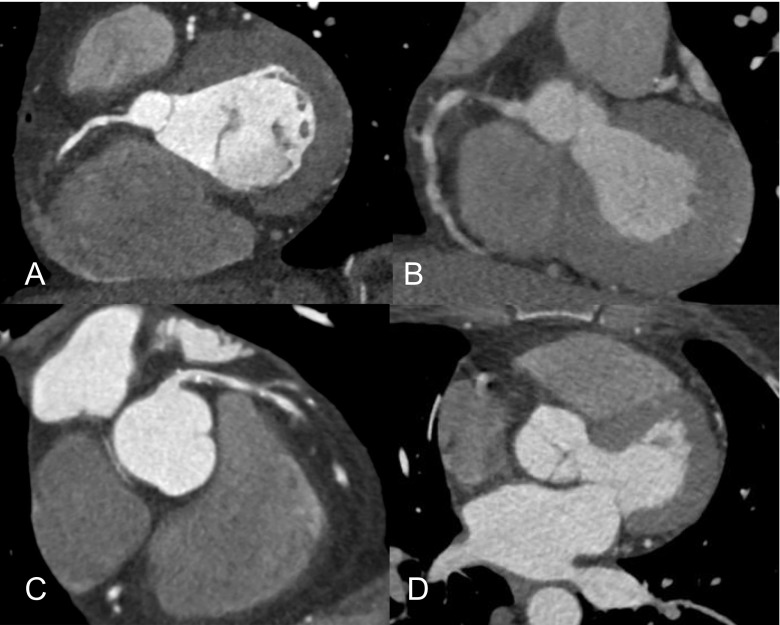
Example of image quality in patient presenting during office hours (**a** and **b**), and outside office hours (**c** and **d**), showing a good image quality, with a good coronary enhancement, no motion or other artefacts of the right coronary artery (**a** – during office hours), and a well-defined lumen with partly calcified and partly non-calcified plaque in the left main coronary artery and left anterior descending (**c** – outside office hours). Poor image quality due to motion artefacts and low contrast enhancement of the right coronary artery are shown (**b** – during office hours). Poor image quality mainly due to major motion artefacts (**d** – outside office hours), made diagnostic evaluation to be deemed impossible

The total quality score per patient was higher for patients presenting during office hours in comparison to patients presenting outside office hours (30.5 [26.0–33.5] vs. 27.5 [19.75–32.0], p=0.043, Table 3). The total per-patient quality score of the proximal coronary segments (segments 1, 2, 5-7 and 11) was higher for patients presenting during office hours (16.0 [14.0–17.0] vs. 15.0 [10.5–17.0], p=0.014). The number of non-evaluable segments was lower for patients presenting during office hours (0 [0–1] vs. 1 [0–4], p= 0.009), as well as the number of non-evaluable proximal coronary segments (0 [0–0] vs. 0 [0–2], p=0.021). An example of image quality is shown in Fig. 2.

**Table 3:** Median quality score

	Office hours (n=89)	Outside office hours (n=29)	p-value
Total quality score of all segments	30.5 [26.0–33.5]	27.5 [19.75–32.0]	0.043
Quality of all proximal segments^a^	16.0 [14.0–17.0]	15.0 [10.5–17.0]	0.014
Total number of unevaluable segments	0 [0–1.0]	1.0 [0–4.0]	0.009
Number unevaluable proximal segments^a^	0 [0–0]	0 [0–2.0]	0.021

Image quality was graded on an ordinal scale ranging from 1 to 3, 1 representing poor image quality due to major artefacts, no diagnostic evaluation possible, 2 artefacts present, but image quality was adequate for diagnostic evaluation, and grade 3, no motion artefacts present, good image quality

^a^Proximal segments included segment 1, 2, 5-7 and 11

### Clinical outcome and downstream testing

From the group of patients presenting during office hours, 22 (25 %) patients were admitted, in comparison to 12 (41 %) patients who presented outside office hours (p=0.085). The overall number of major adverse cardiac events (including all-cause mortality, myocardial infarction and coronary revascularisation) within 30 days after the index visit was low, and comparable between groups (9 (10 %) vs. 3 (10 %), p=0.320)). In addition, there was no difference in the rate of invasive coronary angiography and the rate of coronary revascularisation between patients presenting during and outside office hours (Table 4). The length of stay at the ED was significantly longer for patients presenting outside regular office hours (4.8 [4.0–7.5] vs. 6.4 [5.5–22.9] h, p=0.005).

**Table 4:** Clinical outcome and downstream testing within 30 days after index visit

	Office hours (n=89)	Outside office hours (n=29)	p-value
Admitted to hospital ^a^	22 (25)	12 (41)	0.085
Length of ED stay, hours	4.8 [4.0–7.5]	6.4 [5.5–22.9]	0.005
Major adverse cardiac events	9 (10)	3 (10)	0.320
Invasive coronary angiography^b^	13 (15)	5 (17)	0.749
Coronary revascularisation^b^	8 (9)	2 (7)	0.714

Values are n (%) or median [interquartile range]

^a^Admission to hospital is defined as at least 8 h in hospital

Major cardiac adverse events include all-cause mortality, myocardial infarction and coronary revascularisation. ^b^ Includes procedures at index visit

*ED* emergency department

## Discussion

In this pre-specified sub-analysis of the BEACON trial, we assessed the feasibility of CCTA outside office hours. Our results show that image quality of CCTA outside office hours is slightly lower than during office hours. While no worse clinical outcome was observed after 30 days, patients presenting outside office hours had a longer length of stay and were more likely to be admitted to hospital.

### CCTA performance

To the best of our knowledge, this is the first study to investigate round-the-clock utilisation of CCTA for suspected ACS. We found image quality of CT scans made outside office hours to be sufficient in the majority of patients. There is a small but statistically significant difference in image quality and non-assessable scans in favour of examinations performed during office hours. An explanation for this difference was not immediately evident from the clinical characteristics, type of scanner or acquisition parameters, which were largely comparable between the two groups. However, there was a trend towards higher heart rates in patients presenting outside office hours, which might partly explain the difference in heart rate. A relative small sample size may have obscured any existing difference in clinical characteristics or acquisition parameters. Also, other unidentified confounders or a combination of factors may explain the lower image quality of CT scans acquired outside office hours. While not investigated, experience of the technician and workflow pressure in the emergency ward during off-hours could have played a role as well.

### Differences in clinical outcome

Patients presenting outside office hours had a longer length of stay with the tendency to be admitted more often, which is consistent with previous observations [8, 9]. Logistic reasons, such as the accessibility to testing and staffing, next to unfavourable clinical characteristics are suggested as possible reasons for this difference. In our study, the clinical profiles were unexpectedly comparable between patients presenting outside or within office hours. Their short-term prognosis, expressed in major adverse cardiac events, was also not different. While the difference was small, lower image quality and subsequent less reliability of CCTA outside office hours might have contributed to a longer hospital stay. Also, logistic reasons and the inclination of physicians to admit patients more easily during night hours, rather than clinical profiles, may also play a role.

### 24/7 implementation of CCTA at the emergency department

While CCTA is becoming an accepted diagnostic tool in the workup of low- to intermediate-risk patients presenting with suspected acute coronary syndrome, round-the-clock performance of CCTA at the emergency department has not yet been widely implemented [12]. CCTA is still one of the more demanding CT examination that requires state-of-the-art scanner technology, sufficiently trained staff and time for preparation and optimisation of the procedure. Also, it is important that CT readers at the ED are well trained in assessing CCTA. These conditions are difficult to achieve 24 hours per day, 7 days per week, which is why most centres opt to limit the cardiac CT service to regular office hours. Some of the current barriers to full-time implementation may be overcome in the future with further improved technology, lowering the complexity and lessening the need for premedication, as well as remote expert reading.

### Limitations

There are some study limitations that need to be addressed. The current sub-analysis comprises a relatively small number of patients from a single centre. The proportion of patients enrolled outside office hours (25 %) was lower than expected. Screening of all potential study candidates by the medical team may not have been concordant during regular hours and shifts. Quality of CCTA depends on the available technology and personnel. Therefore, extrapolation of our results to other centres may be limited. Larger studies with patients enrolled in different centres are needed to thoroughly address the performance of CCTA outside office hours.

## Conclusion

Image quality of CCTA outside office hours in the diagnosis of suspected ACS is diminished.

## Abbreviations

ACS: Acute coronary syndrome
AHA: American Heart Association
CCTA: Coronary CT angiography
BEACON: Better Evaluation of Acute Chest Pain with Computed Tomography Angiography
ED: Emergency department
DLP: Dose length product
mSv: Millisieverts
